# Preconception care for diabetic women for improving maternal and fetal outcomes: a systematic review and meta-analysis

**DOI:** 10.1186/1471-2393-10-63

**Published:** 2010-10-14

**Authors:** Hayfaa A Wahabi, Rasmeia A Alzeidan, Ghada A Bawazeer, Lubna A Alansari, Samia A Esmaeil

**Affiliations:** 1 Chair of Evidence-Based Healthcare and Knowledge Translation, King Saud University, Riyadh, Saudi Arabia

## Abstract

**Background:**

Preexisting diabetes mellitus is associated with increased risk for maternal and fetal adverse outcomes. Despite improvement in the access and quality of antenatal care recent population based studies demonstrating increased congenital abnormalities and perinatal mortality in diabetic mothers as compared to the background population. This systematic review was carried out to evaluate the effectiveness and safety of preconception care in improving maternal and fetal outcomes for women with preexisting diabetes mellitus.

**Methods:**

We searched the following databases, MEDLINE, EMBASE, WEB OF SCIENCE, Cochrane Library, including the CENTRAL register of controlled trials and CINHAL up to December 2009, without language restriction, for any preconception care aiming at health promotion, glycemic control and screening and treatment of diabetes complications in women of reproductive age group with type I or type II diabetes. Study design were trials (randomized and non-randomized), cohort and case-control studies. Of the 1612 title scanned 44 full papers were retrieved of those 24 were included in this review. Twelve cohort studies at low and medium risk of bias, with 2502 women, were included in the meta-analysis.

**Results:**

Meta-analysis suggested that preconception care is effective in reducing congenital malformation, RR 0.25 (95% CI 0.15-0.42), NNT17 (95% CI 14-24), preterm delivery, RR 0.70 (95% CI 0.55-0.90), NNT = 8 (95% CI 5-23) and perinatal mortality RR 0.35 (95% CI 0.15-0.82), NNT = 32 (95% CI 19-109). Preconception care lowers HbA1c in the first trimester of pregnancy by an average of 2.43% (95% CI 2.27-2.58). Women who received preconception care booked earlier for antenatal care by an average of 1.32 weeks (95% CI 1.23-1.40).

**Conclusion:**

Preconception care is effective in reducing diabetes related congenital malformations, preterm delivery and maternal hyperglycemia in the first trimester of pregnancy.

## Background

Diabetes mellitus (DM) is a global public health problem with expected 300 million diabetics by the year 2030 worldwide [1]. In many areas around the globe including the West as well as many developing and Middle Eastern countries, diabetes has become a major health burden affecting young adults and women in their reproductive age [2,3].

Despite improved access and quality of antenatal care, women with pre-gestational diabetes and their fetuses are at increased risk of developing serious complications compared with the non-diabetic pregnant women, including spontaneous abortion, preterm labor, hypertensive disorders, and delivery by cesarean section [4,5]. In the recent report of The Confidential Inquiry into Maternal and Child Health (CEMACH) from England, Wales and Northern Ireland, the perinatal mortality in mothers with type 1 and type 2 DM is four times higher and the risk of congenital malformation in the babies of women with diabetes is nearly three times greater [4]. Similar reports from North America showed no significant improvement in fetal and neonatal outcomes of women with pre-gestational diabetes between 1988 and 2002 [6] despite the Saint Vincent Declaration in 1989 which sets a healthcare goal to improve the outcome of pregnancies in diabetic women [7].

Similar reports from the Middle East showed higher rate of perinatal mortality in diabetic as compared to non-diabetic women [8].

Many of the complications of DM during pregnancy can be prevented by optimizing maternal health in the preconception period. Glycemic control is one of the most important aspects of preconception care (PCC) [9]; however other aspects such as folic acid supplementation, smoking cessation, screening and treatment of diabetes complications and discontinuing teratogenic medication, are as important for improving maternal and fetal outcomes [10].

We carried out a systematic review to assess the effectiveness and safety of PCC in improving maternal and fetal outcomes for women with preexisting type 1 or type 2 DM.

## Methods

### Type of studies

We included in this review randomized trials (including cluster and quasi randomized studies) and cohort and case control studies, comparing the frequency of maternal and fetal adverse outcomes in diabetic women who received PCC with those who did not receive PCC.

### Type of participants

Women of reproductive age with preexisting type 1 or type 2 diabetes mellitus who were not pregnant at the time of intervention.

### Type of intervention

For the purpose of this review PCC is defined as the following either as sole intervention or in combination

1. Glycemic control by insulin and/or diet aiming at fasting blood glucose ≤5.7 mmol/l or/and postprandial blood glucose ≤7.8 mmol/l and/or glycosylated hemoglobin A (HbA1C) ≤7.0%)

2. Women counseling and/or education about diabetes complications during pregnancy, the importance of glycemic control and self monitoring of blood glucose level.

3. Preconception screening and treatment of complications of diabetes

4. The use of contraception until optimization of glycemic control is achieved

5. Intake of multivitamin or folic acid in the preconception period.

### Type of outcome

#### Maternal outcomes

1. HbA1C level in the first trimester.

2. Gestation age at the time of the first visit to antenatal care clinic (booking visit).

3. Pregnancy complications including spontaneous abortion, termination of pregnancy due to congenital malformations, polyhydramninos, pre-eclampsia, preterm delivery (before 37 completed weeks from the last menstrual period) and induction of labour due to complication of diabetes.

4. Delivery by cesarean section or instrumental delivery.

5. Maternal hypoglycemia in the first trimester or any other adverse effect reported by the authors.

#### Neonatal outcomes

1. Congenital malformation related to maternal diabetes

2. Total mortality (stillbirth and neonatal death).

3. Birth trauma

4. Admission to neonatal intensive care unit (NICU).

5. Respiratory distress syndrome (RDS)

6. Macrosomia (birth weight ≥4 kg for term infants or birth weight ≥90^th ^percentile for the gestation age)

7. Small for gestational age (SGA) (birth weight below the 10^th ^percentile for the gestational age).

8. Shoulder dystocia.

### Exclusion criteria

We excluded from this review reports which are not of comparative design and reports of conference proceedings or abstracts when there is no complete description of the trial or study.

### Search strategy

The search strategy was developed in consultation of an information retrieval specialist. We searched the following databases, MEDLINE (1966-December 2009), EMBASE (1980-December2009), WEB OF SCIENCE (Science citation index-1970-December 2009), Cochrane Library up to the latest issue 2009, including the CENTRAL register of controlled trials and CINHAL (Cumulative Index to Nursing & Allied Health 1982 -December 2009). (For full search strategy see Additional file 1: Appendix 1)

We reviewed the reference list of all relevant studies for any potential study not retrieved by the search strategy. Unpublished reports were not actively sought and there was no language limitation.

### Identification of included studies

All titles and abstracts retrieved by the electronic search were screened independently by three reviewers and the studies which clearly did not meet the inclusion criteria were excluded. Copies of the full text of potentially relevant studies and trials were obtained and their eligibility was assessed independently by two reviews. Differences between reviewers were resolved by discussion or by consulting a third reviewer.

### Data extraction and studies assessment

Three authors extracted data from the included studies using a designed form. The accuracy of the extracted data was checked by two other reviewers.

The Newcastle Ottawa Scale (NOS) was used for the assessment of cohort, case control studies and non-randomized trials [11]. Risk of bias in each study, was assigned according to the number of items on the NOS judged to be inadequate. We considered low risk of bias when one item is inadequate, medium risk of bias when up to three items are inadequate and high risk of bias when more than three items are inadequate. Data analysis was carried out with the use of Review Manager Software 5.0(Cochrane Collaboration, Oxford, United Kingdom).

Meta-analysis was performed for studies with similar design and type of intervention, which we assessed to be at medium or low risk of bias using the fixed effect model. Heterogeneity is considered high when I^2 ^>50% and explanation was attempted however subgroup analysis was not possible in most of the cases due to the small number of studies. Pooled data were presented as risk ratio (RR) with 95% confidence intervals (95% CI) for dichotomous outcomes and as the means difference with 95% confidence intervals for continuous outcomes.

## Results

**Figure 1:**
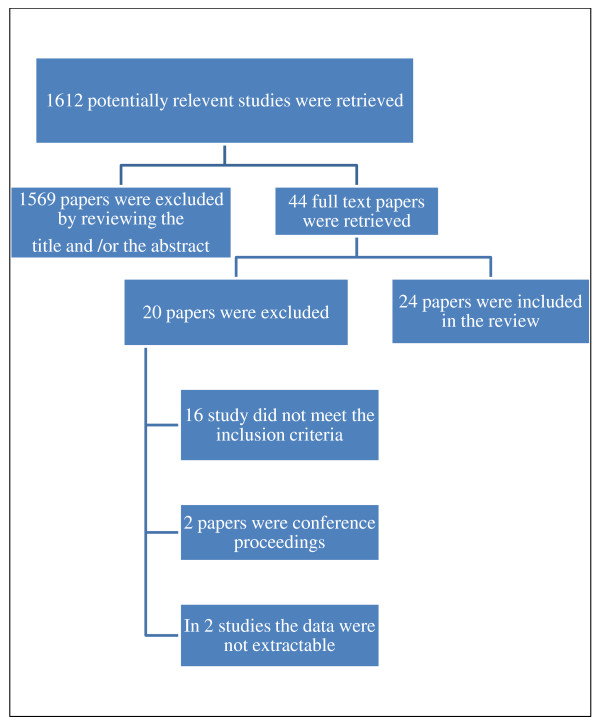
Process of selection of the studies for the systematic review.

The search retrieved 1612 potentially relevant titles of which the full papers of 44 relevant reports were reviewed (Figure 1). A total of 24 reports of 20 studies were included in this review [10,12-34]. (Three articles described the same cohort study with two interim [17,18] and one final report [19], one study reported the outcomes for the same cohort in two articles [10,29] and two articles report the outcomes of one cohort with one interim [31] and one final report [28]).

Twenty studies were excluded, 16 of them were excluded because they did not meet the inclusion criteria, 2 reports were of conference proceedings and in 2 studies data were not extractable (Additional file 1: Appendix 2).

Of the included studies, only one was a controlled trial, 11 studies were prospective cohort studies, 7 studies were retrospective cohort studies and one was a case control study (Tables 1-4).

**Table 1:** Characteristics of included Prospective Cohort Studies

Study/Year of Publication Reference (country)	Participants	Intervention	Outcome	Risk of Bias (Notes)
**Garcia-Patterson 1997 **[20]**(Spain)**	66 participants with type I and type II who attended the preconception clinic and 119 participants with type I and type II diabetes who did not.	PCC included intensive insulin therapy, self-monitoring of blood glucose and dietary advice	The HA1C was significantly better in the PCC group than for the NPCC group (*p *= 0.01). The rate of cesarean section was higher in the PCC group than the NPCC. No differences were observed in abortion, Pre-eclampsia and preterm labor. Small for gestation age was more in the NPCC.	Medium (The baseline characteristics in relation to the vasculopathy are different. No blinding for the outcome assessment).

**Herman 1999 **[22]**(USA)**	24 women with type I diabetes who attended the preconception clinic, and 74 women with type I diabetes who did not attend the preconception clinic.	PCC included education, counseling, glycemic control, and assessment of complications of diabetes such as nephropathy and retinopathy	Women who had PCC had significantly more spontaneous abortion, significantly lower level of HA1C at booking and throughout pregnancy and significantly heavier infants at birth than NPCC group (*p *< 0.05). There was no significant difference between the two groups in the frequency of infants with congenital malformations, gestation age at delivery or frequency of neonatal admission to the intensive care unit.	High (The study was not designed to assess the clinical outcomes of the preconception care but the differences in the socio-demographic features between the groups who attend the preconception care and those who did not. The target level for the glycemic control was not clear and the absolute level of Hb A1C at booking and all through pregnancy for the study and the control groups was not mentioned)

**Jaffiol 2000 **[23]**(France)**	21 IDDM attended the pre-conception care and 40 did not attend	PCC included education, glycemic control self monitoring of blood glucose and Contraception	The investigated outcomes included polyhydramninos, pre-eclampsia, premature deliver, rate of cesarean section, rate of spontaneous and therapeutic abortion, perinatal and neonatal mortality, neonatal hypoglycemia and birth trauma. Significant reduction in the total fetal loss, neonatal mortality and congenital malformations (*p *< 0.05), the level of maternal HA1C in the 1^st ^trimester (*p *< 0.05) and total adverse obstetrics complications (*p *< 0.05)	Low (good report, clear intervention description, the comparative groups received same antenatal intervention. No blinding for outcome assessment)

**Jensen 1986 **[24]**(Denmark)**	9 women with insulin dependent diabetes had preconception care and 11 women with insulin dependent diabetic who did not receive preconception care.	PCC included continuous insulin infusion initiated 2 months prior to conception	No significant difference in congenital malformations and HA1C level, between the two groups	High (small number of study and control group, many differences in the baseline characteristics in the severity of diabetes, 5 of the 11 control women were treated in the diabetic clinic in the hospital before pregnancy so they knew about the importance of glycemic control both groups have the same HA1C levels in early pregnancy)

**Kitzmiller 1991**[25]**(USA)**	84 women in preconception care and 110 women had no preconception care	PCC included glycemic and dietary control education, exercise and contraception.	The frequency of congenital abnormalities in the PCC group was 1.2% compared to 10.9% in the NPCC group (*p *< 0.05). There were 12 spontaneous abortion in the preconception care group and 14 in the group who received no preconception care.	Low (good report clear methodology)

**Rosenn 1991**[26]**(USA)**	28 women in the preconception group and 71 in the control group	PCC included dietary advice and glycemic control	HA1C concentration in the PCC group was lower than in the NPCC group (*p *< 0.0008). Spontaneous abortion rate was lower (*p *< 0.04) and there was no congenital malformations in either group.	Medium (52% of preconception care patients dropped out, no blinding in the assessment of the outcome)

**Temple 2006a **[10]**2006b **[29]**(UK)**	110 women with type I diabetes attended the preconception care clinic and 180 women with type I diabetes did not attend the preconception care clinic	PCC included: Glycemic control, folic acid supplementation, smoking cessation, education.	There was significant improvement in the outcome between the PCC group and the NPCC group in the rate of spontaneous abortion (*p *< 0.056) and in the rate of preterm delivery (*p *< 0.02). The rate of congenital malformations was lower in PCC group compared to the NPCC group (*p *< 0.065). the adverse outcome including malformations, still birth and neonatal death were significantly more in the latter group than the former one (*p *< 0.026)	Low (Baseline characteristics in both groups were similar; the prospective nature of the study ascertained the completeness of the follow up, the completeness of the baseline and the outcome data. Use of appropriate statistical tests such as logistic regression analysis confirmed the association between the preconception care and outcomes).

**Willhoite 1993**[30]**(USA)**	62 women with either type I or type II diabetes who received preconception counseling and 123 women with either type I or type II diabetes who did not receive preconception counseling	PCC included counseling by health professional the control group received no counseling.	PCC group had significantly less perinatal mortality than the NPCC group (OR3.9 CI 1.2-13.9) and insignificantly less congenital malformations (OR 4.2 CI 0.5-29.7)	High (Base line characteristics of the two groups were significantly different in age, duration of diabetes and smoking all are confounding factors for the outcomes. The two groups did not receive the same antenatal intra-partum and postnatal care. The assessor of the congenital malformation was not blinded)

**Boulot 2003 **[33]**(France)**	172 women with either type I or type II diabetes who received PCC and 260 women with either type I or type II diabetes who did not receive PCC	PCC included education, assessment of diabetes complications glycemic control self monitoring of blood glucose and Contraception	PCC group had significantly less perinatal mortality than the NPCC group, (*p *< 0.005) for type 1 diabetics and significantly less congenital malformations, (*p *< 0.005) for type 1 diabetics	Low (cases and control were well defined and comparable, selection bias is unlikely as consecutive cases were enrolled, the prospective nature of the study ascertained the completeness of the follow up, the completeness of the baseline and the outcome data)

**Galindo 2006 **[32]**(Spain)**	15 women with pre-existing diabetes received PCC and 112 women with pre-existing diabetes did not receive PCC.	PCC included education, glycemic control self monitoring of blood glucose	The frequency of congenital abnormalities in the PCC group was 3/15 compared to 14/112 in the NPCC group. There was 1 spontaneous abortion in the PCC group and 9 in the group who received no PCC.	Low (cases and control were well defined and comparable, selection bias is unlikely as consecutive cases were enrolled, the prospective nature of the study ascertained the completeness of the follow up, the completeness of the baseline and the outcome data)

**Garcia Ingelmo 1998 **[34]**(Spain)**	12 women with pre-existing diabetes received PCC and 12 women with pre-existing diabetes did not receive PCC	PCC glycemic control.	The frequency of congenital abnormalities in the PCC group was 3/12 compared to 2/12 in the NPCC group. In the PCC 6/12 neonates were macrosomic while 4/12 were macrosomic in the NPCC group. HbA1c was significantly lower in the first trimester in the PCC group compared to the NPCC group , (*p *< 0.01)	High (Both the study population and the control were not representative of the general diabetic population with frequency of diabetic vascular complications approaching 50%. The PCC components were not defined neither the target blood glucose)

Key: HbA1c = Glycosylated Hemoglobin A, PCC = Preconception Care, NPCC = No Preconception Care, OR = Odd Ratio, IDDM= Insulin depended Diabetes Miletus, CI = Confidence Interval

**Table 2:** Characteristics of included retrospective cohort studies

Year of Publication (country)	Participants	Intervention v comparison	Outcome	Risk of Bias
**Dicker 1988 **[15]**(Israel)**	59 IDDM women attended a pre-conception clinic compared to 35 pregnant women who did not attend	PCC included: insulin and dietary glycemic control, advice on contraception and screening for diabetes complications	PCC group had significantly lower HA1C at the first trimester (*p *< 0.001) and significantly lower rate of spontaneous abortion (*p *< 0.001) compared to the NPCC group.	Low (Clear description of participants and intervention, noted confounding factors and well presented results. There was significant difference between the two groups in the diabetes complications before intervention)

**Dunne 1999 **[16]**(UK)**	47 women with IDDM 12 of them attended preconception care clinic and 35 women did not.	PCC included assessment of diabetes complications and glycemic control	The PCC group had significantly lower level of HA1C level compared to the NPCC group (*p *< 0.008). There were no congenital malformations in both groups. The cesarean section rate, the macrosomia rate and the small for gestation age were similar between the two groups	Medium (Due to the audit nature of the report there is no clear description of the intervention, some important confounders were not addressed such as White's classification and the outcome assessment was not blinded )

**Damm 1989 **[14]**(Denmark)**	197 attended PCC and 61 didn't attend	PCC included: contraception and glycemic control.	The rate of congenital malformations was significantly lower in the PPC group 1.0% than the NPPC group 8.2%, (*p *< 0.01). No significant difference in the level of HA1C during the first trimester between the two group	High (unclear description of the participants, the intervention and the outcome, the data of the preconception care were a subset of from different periods of the study)

**Goldman 1986 **[21]**(Israel)**	44 women with type I diabetes attended the preconception clinic and 31 women with type I diabetes did not attend	PCC included assessment of diabetic complications, Contraception advice, Glycemic control and dietary advice	The NPCC group had significantly shorter duration of pregnancy (*p *< 0.05) significantly heavier mean birth weight (*p *< 0.05) than the PCC group. The two groups were similar in neonatal hypoglycemia, hypocalcaemia and respiratory distress syndrome	Low (Clear description of participants and intervention, noted confounding factors and well presented results. There was significant difference between the two groups in the diabetes complications before intervention)

**Fuhrmann 1986 **[19]**& 1984 **[18]**& 1983 **[17]**(Germany)**	620 pregnant women with insulin dependent diabetes,183 received pre-pregnancy care 437 women did not	PCC included: short hospitalization every 3 month until conception, education, self monitoring of blood glucose, assessment and treatment of diabetes complications and glycemic control	PCC group had significantly lower rate of congenital malformations 1.1% compared to the NPCC group 7.0% (*p *< 0.01)	Medium (Well described intervention, no blinding for the outcome, no description of the possible confounding factors)

**Rowe 1987 **[27]**(UK)**	21 IDDM 14 received preconception care and 7 did not	PCC included Glycemic control, counseling and blood glucose self monitoring	The PCC group had significantly better initial HA1C level (*p *< 0.0001), and lower mean birth weight (*p *< 0.05)	High (Unclear description of the participants, no description of possible confounding factors, no blinding in assessment of the outcome, small group, high target of HbA1C 5-9%)

**Steel 1990 **[28]**& 1982 **[31]**(UK- Scotland)**	143 IDDM women attended the preconception care clinic and 96 IDDM women did not attend	PCC included: education, glycemic controlled and contraception	PCC group had lower initial HbA1C as compared to NPCC group (*p *< 0.0001) and lower rate of congenital mal formations (*p *< 0. 005) , maternal hypoglycemia was significantly common in the PCC group than the NPCC (*p *< 0. 001)	Medium (Good description of interventions, contamination of the control who might know about the usefulness of the and the outcome assessment was not blinded )

Key: HbA1C = Glycosylated Hemoglobin A, PCC = Preconception Care NPCC = No Preconception Care, OR = Odd Ratio, IDDM = Insulin depended Diabetes Miletus

### Assessment of the methodological quality of the included studies

The cohort studies included in this review (Table1&2) had adequate description of participants including description of some confounding factors such as the frequency of renal and vascular complications of diabetes between the PCC group and the control group. However all studies did not address the effect of the presence of confounding factors on the outcomes except for 2 reports which used regression analysis to evaluate the effectiveness of the PCC [10,29].

In most of the cohort studies blinding of the control group was adequate because they were recruited after pregnancy when they attended for antenatal care, except for 2 studies [24,28], in which inadequate blinding of the control group cannot be excluded because they were informed about the importance of the PCC and were invited to attend. All participants received the same antenatal and post natal care except for one study [24] where participants were followed up in different health settings.

All cohort studies had adequate follow up for participants except for one study in which 52% of the PCC group were lost to follow up [26]. The assessors of the outcomes were not blinded to the participants' allocation except in one study [25].

Some of the studies at high risk of bias were initially designed to assess aspects of PCC other than its effectiveness in improving maternal and fetal outcomes, hence the poor methodological design when assessed with the NOS [16,22,30].

PCC in all the cohort studies included control and self monitoring of blood glucose except for one which was designed to examine the effectiveness of preconception counseling on fetal and neonatal outcomes [30]. In addition to glycemic control, 4 studies included screening and treatment of complications of diabetes in the PCC program [16,19,21,22]. Only one cohort study (two reports) had comprehensive PCC program including, control and self monitoring of blood glucose, folic acid supplementation, smoking cessation advice and discontinuation of teratogenic drugs [10,29].

One case-control study was included in this review [13] (Table 3). It examined the effectiveness of multivitamin supplementation in the preconception period in preventing diabetes related congenital abnormalities. The study is at medium risk of bias due to possibility of recall bias during the interview of the mothers and the possibility that interviewers were not blinded to the outcome.

**Table 3:** Characteristics of included case-control studies

Study/Year of Publication (country)	Participants	Intervention	Outcome	Risk of Bias/Notes
**Correa 2003 **[13]**USA**	Cases were 3278 Infants with congenital malformations related to diabetes. Controls were 3029 infants without congenital malformations. Maternal diabetes and intake of multivitamin were evaluated as a risk factors for congenital malformations	PCC included the use of multivitamin for 3 month before conception	The risk of congenital malformations related to diabetes was limited to infants of f diabetic mothers who had not taken multivitamin (OR 3.39 95% CI 1.79-8.63). Mother who had taken multivitamin had no increase risk of congenital malformations related to diabetes (OR 0.15 95% CI 0.00-1.99)	Medium (clear definition and selection of cases and controls, and outcomes, clearly defined outcome, not clear if the interviewers were blinded to the outcome, recall bias cannot be excluded during the interviews)

Key: OR= Odd Ratio, CI= Confidence Interval

One trial was included in this review [12] (Table 4). The design of the trial was not clear as authors reported it as a randomized trial but the method for randomization was not described. There was no allocation concealment and lack of blinding introduced bias because both groups were aware of the importance of the glycemic control and the complications of diabetes during pregnancy.

**Table 4:** Characteristics of included controlled trials

Study/Year of Publication (country)	Participants	Intervention v comparison	Outcome	Risk of Bias/Note
**Pregnancy outcome in Diabetes control and complication trial research group 1996 **[12]**(USA)**	187 had preconception intensive insulin therapy and 83 did not.	PCC included glycemic control and dietary advice.	There were 26 spontaneous abortion in the PCC group and 16 in the NPCC group. One still birth in the PCC group and 3 in the NPCC. Congenital mal formations were 5in the PCC group and 4 in the NPCC group. No differences on neonatal morbidity or maternal morbidity. Mean HbA1C in PCC group =7.4 ± 1.3 and in NPCC = 8.8 ± 1.7	High (Unclear report of the outcome, the control group was aware of the importance of glycemic control and was repeatedly advised to change into intensive therapy when planning pregnancy. So intervention was not restricted to the preconception group. No specific target level of the blood sugar was stated for the preconception group)

Key: HbA1C = Glycosylated Hemoglobin A, PCC = Preconception Care, NPCC = No Preconception Care.

Only 2 studies in this review evaluated maternal hypoglycemia, as an adverse effect of PCC [10,28].

### Outcome of PCC

**Figure 2:**
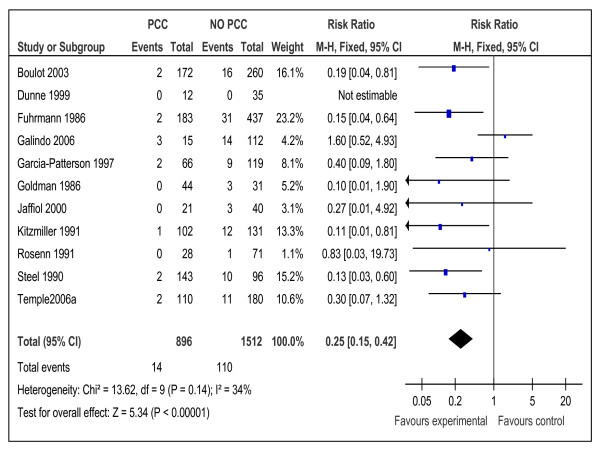
**Risk ratio for congenital malformations from 11 studies of women with preexisting diabetes mellitus who did or did not receive preconception care.** PCC= the group who received preconception care; NPCC= the group who did not received preconception care; CI= Confidence intervals.

**Figure 3:**
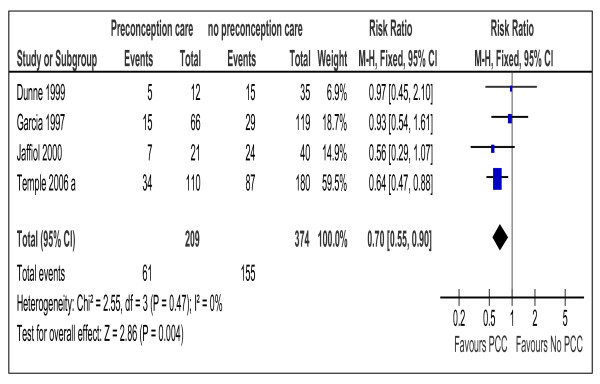
**Risk ratio for preterm delivery from 4 studies of women with preexisting diabetes mellitus who did or did not receive preconception care.** PCC= Preconception care; NPCC= No preconception care; CI= Confidence intervals.

**Figure 4:**
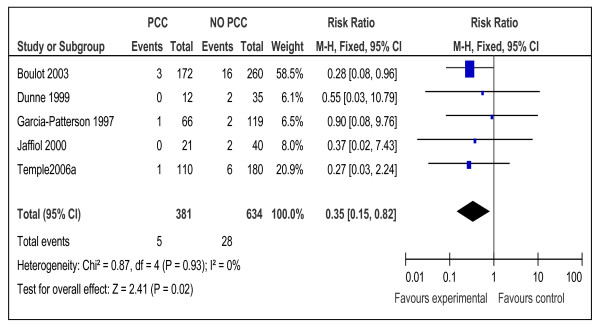
**Risk ratio for perinatal mortality from 5 studies of women with preexisting diabetes mellitus who did or did not receive preconception care.** PCC= the group who received preconception care; NPCC= the group who did not received preconception care; CI= Confidence intervals.

**Figure 5:**
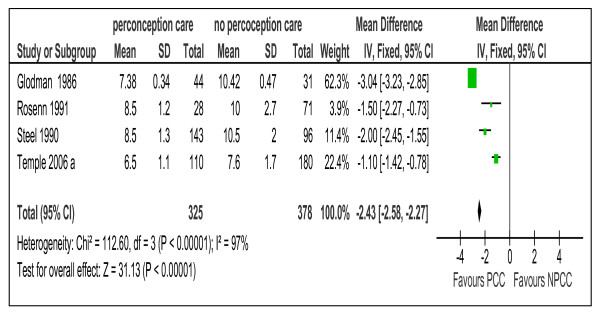
**First trimester mean value of glycosylated hemoglobin from 4 studies of women with preexisting diabetes mellitus who did or did not receive preconception care.** PCC= Preconception care; NPCC= No preconception care; CI= Confidence intervals

**Figure 6:**
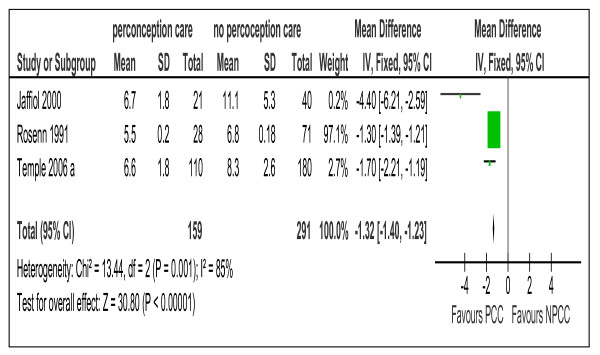
**The mean gestation age at the time of the first antenatal visit from 3 studies of women with preexisting diabetes mellitus who did or did not receive preconception care.** PCC= Preconception care; NPCC= No preconception care; CI= Confidence intervals.

**Figure 7:**
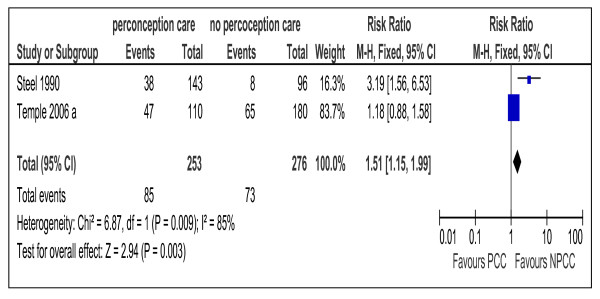
**Risk ratio for maternal hypoglycemia from 2 studies of women with preexisting diabetes mellitus who did or did not receive preconception care.** PCC= Preconception care; NPCC= No preconception care; CI= Confidence intervals.

Similarity of participants, intervention, and outcomes in addition to the score of low or medium risk of bias, made meta-analysis possible for12 cohort studies [10,15,16,19-21,23,25,26,28,32,33] with 2502 participants (Tables 1, 2&5 and Figures 2, 3, 4, 5, 6, and 7). Both dichotomous and continuous data were pooled but only when standard deviation and similar units were available for continuous data. Studies which were at high risk of bias or of a design other than cohort were excluded from the meta-analysis.

Meta-analysis suggested that preconception care is effective in reducing congenital malformation, RR 0.25 (95% CI 0.15-0.42), NNT17 (95% CI 14-24), preterm delivery, RR 0.70 (95% CI 0.55-0.90), NNT= 8 (95% CI 5-23) and perinatal mortality RR 0.35 (95% CI 0.15-0.82), NNT= 32 (95% CI 19-109) (Figures 2, 3, and 4).

Meta-analysis of 5 trials show that PCC lowers HbA1C in the first trimester of pregnancy by an average of 2.43% (95% CI 2.27-2.58) and while there is high heterogeneity (I^2^= 97%) this variation is in the size of the effect rather than the direction (Figure 5).

Women who received PCC booked earlier during pregnancy for antenatal care compared to women who did not, by an average of 1.32 week (95% CI 1.4-1.23) (Figure 6)

The evidence did not support the effectiveness of the PCC in reducing, spontaneous abortion, pre-eclampsia, cesarean delivery, macrosomia, RDS, SGA and neonatal hypoglycemia (Table 5 and Additional file 1:Appendix 3).

**Table 5:** Pooled estimates effect of preconception care

Dichotomous outcomes of preconception care	No of studies [references]	Risk Ratio (95%Confedance interval)
Congenital malformation	11 [10,16,19-21,23,25,26,28,32,33]	0.25(0.15,0.42)

Perinatal Mortality	5 [10,16,20,23,33]	0.35(0.15,0.82)

Macrosomia	3 [10,20,23]	1.03(0.81,1.30)

Cesarean Section	5 [10,16,20,21,23]	1.08(0.96,1.22)

Preterm Delivery	4 [10,16,20,23]	0.7 (0.55,0.90)

Pre-eclampsia	3 [10,20,21]	0.92(0.62,1.35)

Neonatal Hypoglycemia	3 [20,21,23]	0.65(0.39,1.08)

Maternal Hypoglycemia	2 [10,28]	1.51(1.15,1.99)

Spontaneous Abortion	7 [10,15,20,23,25,26,32]	0.78(0.55,1.11)

Respiratory Distress Syndrome	3 [20,21,23]	0.55(0.26,1.16)

Small for Gestation Age	2 [20,23]	0.26(0.05,1.41)

**Continuous outcomes**	**Number of studies ( references)**	**Means difference (95% CI)**

The difference in the level of glycosylated Hemoglobin A1c	4 [10,21,26,28]	2.43(2.27,2.58)

The difference in gestational age at first visit to antenatal care	3 [10,23,26]	1.32 (1.23, 1.40)

The use of multivitamins in the preconception period as a sole intervention, was evaluated by one case control study [13] and was found not to be effective in reducing the rate of congenital malformations (Odd Ratio (OR) 0.15 95% CI 0.00-1.99).

Similarly one study, at high risk of bias, evaluated the effectiveness of preconception counseling, as a sole intervention, in improving fetal and neonatal outcomes, showed improvement in total mortality (still birth and neonatal death) and the rate of congenital malformation [30].

Hypoglycemia as adverse effect of PCC was evaluated by two studies [10,28]. Meta-analysis of the pooled data did not show difference between the PCC and the control group (Figure 7).

Data were not available for the evaluation of the effects of PCC on polyhydramninos, termination of pregnancy for congenital malformations, induction of labor, birth trauma, shoulder dystocia and admission to NICU.

## Discussion

Our systematic review of the effectiveness of PCC in the improvement of maternal and fetal outcomes, found sufficient evidence to support its implementation in practice.

The nature of the intervention lent strength to the observational studies by avoiding certain biases known to occur in such study designs. Lack of allocation concealment and blinding of participants were avoided by recruiting the intervention and the control groups at different times during the course of the study (preconception period and antenatal period). Due to the relatively short duration of the pregnancy, attrition bias was noted in only one study, [26] all other studies had complete follow up of both groups. However the problem of confounding factors such as smoking, maternal age, parity and vascular complications of diabetes, was noted by most of the studies but only one study used the appropriate statistical test to quantify the effect of the PCC apart from the confounders [10].

The homogeneity of the participants, the intervention and the outcomes gives confidence in the estimated effects of the PCC from the pooled data.

The effectiveness of PCC in reducing congenital malformations is impressive (Table 5 and Figure 2) and has practical implication considering the recent report of the CEMCH [4] which showed that congenital malformations rate in infants of diabetic mothers in England, Wales and Northern Ireland is more than twice the background population rate. This finding is also of a paramount importance to many communities in the Middle East [35], North Africa [36] and some communities in Asia [37] where the burden of congenital malformation is very high due to many causes including maternal diabetes.

The effect of PCC in reducing the rate of congenital malformations reflected positively on its effect in reducing the perinatal mortality among women who utilized the care (Figure 4). This effect addresses a major health problem of four folds increase in the perinatal mortality in mothers with preexisting diabetes when compared to the general population [38]

The meta-analysis supported the effectiveness of the PCC in reducing the rate of preterm delivery (Table 5 and Figure 3). We believe that effect would have been larger if data were available for very preterm delivery ≤34 weeks of gestation when the effect of the preconception rather than the antenatal care is evaluated as demonstrated by one study [10].

Maternal hyperglycemia during the period of organogenesis is known to be associated with congenital malformations [39,40]. The analysis of the pooled data in this review suggested that PCC is effective in reducing the level of HbA1C during the first trimester of pregnancy and hence the risk of congenital malformations (Table 5 and Figure 5).

We were surprised that meta-analysis did not support the effectiveness of PCC in improving the rates of spontaneous abortion (Table 5 and Additional file 1: Appendix 3). We suggest that this result is due to late attendance of the control group for antenatal care by which time some events of spontaneous abortion might have been missed. This suggestion was further supported by meta-analysis of the gestation age at first visit for the PCC and the control groups, (Figure 6) which showed significant difference between the two groups.

In this review one case control study addressed the effectiveness of multivitamins supplementation in the preconception period, as an isolated intervention, in reducing the rate of congenital malformations[13]. The role of folic acid and multivitamins in the prevention of some congenital malformation is well documented [41]. However all other studies included in this review, except for one recent report [10], did not include multivitamin or folic acid in their program of PCC, which supports an expectation of larger effect of PCC in improving fetal and neonatal outcomes if folic acid or multivitamin supplementation becomes an integral part of that care.

Another isolated preconception intervention proved to be effective in improving fetal and neonatal outcomes, is women counseling, an intervention evaluated by only one study [30]

Other outcomes which did not improve by PCC, such as pre-eclampsia, cesarean delivery and macrosomia (Table 5 and Additional file 1: Appendix 3), might be related to care during the latter part of pregnancy rather than the preconception period. However few studies were included in the meta-analysis for these outcomes and further larger studies, with more participants might prove the effectiveness of PCC in improving some or all of these outcomes.

Only two studies evaluated maternal hypoglycemia as adverse effect of PCC [10,28] and the pooled data showed no difference between the two groups (Figure 7). Marked heterogeneity might be due to the differences in the target blood glucose level between the two studies.

One study conducted an economic evaluation of PCC and found that it is associated with considerable saving and reduced resources utilization [22] and yet population based studies showed that only 34-38% of eligible women receive PCC [4,30].

We suggest that more research is needed in methods of encouraging diabetic women to utilize PCC.

Our review confirms previous findings by Ray et al [9]. The strength of our review comes from the comprehensive evaluation of the available evidence on the effectiveness and safety of PCC in improving maternal and fetal outcomes together with assessment of wide range of interventions which we considered as PCC and all the possible maternal, fetal or neonatal outcomes which are affected by maternal preexisting DM. However we are aware of the limitation of the observations studies as the sole source of evidence and the inherent bias associated with the design of the cohort studies included in the meta-analysis.

The review carries important implications for practice and research as it highlights the importance of the integration of PCC in the routine care of diabetic women during their reproductive age.

## Conclusion

PCC for women with preexisting type 1 or type 2 DM is effective in improving rates of congenital malformation, perinatal mortality, preterm labour, level of maternal HbA1c in the first trimester of pregnancy and maternal early utilization of antenatal care.

## Competing Interests

The authors declare that they have no conflict of interest.

## Authors' contributions

HW conceived the idea of the review, was responsible for drafting and writing the study protocol and reviewing the search strategy. HW, RZ, GB, LA and SE were responsible for study selection and data extraction. HW and RZ were responsible for quality assessment of studies and data analysis. HW was responsible for writing the final report. LA, HW, RZ, GB and SE reviewed and approved the final manuscript.

## Pre-publication history

The pre-publication history for this paper can be accessed here:

http://www.biomedcentral.com/1471-2393/10/63/prepub

## Supplementary Material

Additional files 1 **Appendices **Appendix 1: Search Strategy (the systematic review search strategy). Appendix 2: Table of excluded studies (description of studies excluded from the review and the reasons for exclusion). Appendix 3: maternal and fetal outcomes not improved by preconception care (Forest Plots of meta-analysis of outcomes which are not improved by preconception care). Click here for file
