# Supplementary material for: Preconception care for diabetic women for improving maternal and fetal outcomes: a systematic review and meta-analysis
Source: BMC Pregnancy Childbirth. 2010 Oct 14;10:63. doi: 10.1186/1471-2393-10-63 (PMC2972233; doi:10.1186/1471-2393-10-63)
Supplement: Additional files 1 — Appendices Appendix 1: Search Strategy (the systematic review search strategy). Appendix 2: Table of excluded studies (description of studies excluded from the review and the reasons for exclusion). Appendix 3: maternal and fetal outcomes not improved by preconception care (Forest Plots of meta-analysis of outcomes which are not improved by preconception care). [file 1471-2393-10-63-S1.DOC]

**Appendix 1**:

**Search Strategy**

**Electronic databases searches.**

We searched the following electronic databases:
-MEDLINE (1966 Dec 2009)
-EMBASE (1980 to Dec 2009)
-WEB OF SCIENCE (Science Citation Index – 1970 to Dec 2009)

-Cochrane Library (up to Dec 2009) – including CENTRAL register of Controlled Trials

-CINHAL (Cumulative Index to Nursing & Allied Health; 1982 to Dec 2009)

**Search Strategy in MEDLINE:**
Exp Diabetes mellitus [Mesh]

1. Pregnancy in diabetics [Mesh]
2. Diabet* ti, ab
3. 1 or 2 or 3
4. Preconception care [Mesh]
5. preconception* AND (service* OR counsel* OR program* OR care OR education* OR clinic*) ti, ab
6. pregestational AND (service* OR counsel* OR program* OR care OR education* OR clinic*) ti, ab
7. periconception AND (service* OR counsel* OR program* OR care OR education* OR clinic*) ti, ab
8. Prepregnancy AND (service* OR counsel* OR program* OR care OR education* OR clinic*) ti, ab
9. Pregnancy in diabetics [Mesh] AND (Hemoglobin A1c ti, ab)
10. Pregnancy in diabetics [Mesh] AND (Hyperglycemia [Mesh] OR blood glucose ti,ab)
11. 5 or 6 or 7 or 8 or 9 or 10 or 11
12. randomized controlled trial [pt]
13. controlled clinical trial [pt]
14. randomized OR randomised ti, ab
15. placebo* ti, ab
16. randomly ti, ab
17. clinical trials as topic [Mesh]
18. intervention* ti, ab
19. evaluat* ti, ab
20. compar* ti, ab
21. Case-control studies [Mesh]
22. Cohort studies [Mesh]
23. retrospective ti, ab
24. prospective ti, ab
25. 13 or 14 or 15 or 16 or 17 or 18 or 19 or 20 or 21 or 22 or 23 or 24 or 25
26. 4 and 12 and 26

**Appendix 2**:

**Table of Excluded Studies**

| **Study /Year of Publication** | **Reasons for Exclusion** |
| --- | --- |
| Charron-Prochownik (2008) [1] | The trial examines outcomes different from the outcomes of the review. |
| Cousins (1991) [2] | Outcomes of PCC group were not compared to the group who did not receive PCC. |
| Delgado Del Rey (2001) [3] | There is no control group to compare outcome with preconception care (PCC) group. |
| Gold (1998) [4] | Data for PCC group could not be extracted |
| Mills (1988) [5] | Data for PCC group could not be extracted. |
| Casele (1998) [6] | This is an observational non comparative study. |
| Miller (1981) [7] | The study was on pregnant participants and not preconception group |
| Hod (1995) [8] | The study reports an intervention on a case series with no control group. |
| Jouatte (1999) [9] | This is an observational non comparative study. |
| Rowe (1988) [10] | The study population includes women with impaired glucose tolerance. Outcome for established diabetic participants who received PCC was not reported separately. |
| McElvy (2000) [11] | The study does not compare the outcomes of the PCC group with those of no PCC group. |
| Pearson (2007) [12] | No comparison on the outcome between PCC group and no PCC group. |
| Dicker (1987) [13] | The study compares two types of preconception care rather than PCC to no PCC. |
| Kinsley (2007) [14] | The study does not compare the outcomes of the PCC group with those of no PCC group. |
| Rodgers (1996) [15] | No comparison on the outcome between PCC group and no PCC group. |
| Unger (2001) [16] | Review and recommendation for management. |
| Garcia (1997) [17] | Conference abstract |
| Forde (2006) [18] | Conference proceeding |
| Kitzmiller (1996) [19] | Review and recommendation for management. |
| Mathiesen (2007) [20] | Data for the outcome of PCC not reported. |

Reference List

1. Charron-Prochownik D, Ferons-Hannan M, Sereika S, Becker D: **Randomized efficacy trial of early preconception counseling for diabetic teens (READY-girls).** *Diabetes Care* 2008, **31:** 1327-1330.

2. Cousins L: **The California Diabetes and Pregnancy Programme: a statewide collaborative programme for the pre-conception and prenatal care of diabetic women.** *Baillieres Clin Obstet Gynaecol* 1991, **5:** 443-459.

3. Delgado del RM, Herranz L, Martin VP, Janez M, Juan Lozano GJ, Darias R *et al*.: **[Effect of preconceptional metabolic control in the course of pregnancy in diabetic patients].** *Med Clin (Barc )* 2001, **117:** 45-48.

4. Gold AE, Reilly R, Little J, Walker JD: **The effect of glycemic control in the pre-conception period and early pregnancy on birth weight in women with IDDM.** *Diabetes Care* 1998, **21:** 535-538.

5. Mills JL, Simpson JL, Driscoll SG, Jovanovic-Peterson L, Van AM, Aarons JH *et al*.: **Incidence of spontaneous abortion among normal women and insulin-dependent diabetic women whose pregnancies were identified within 21 days of conception.** *N Engl J Med* 1988, **319:** 1617-1623.

6. Casele HL, Laifer SA: **Factors influencing preconception control of glycemia in diabetic women.** *Archives of Internal Medicine* 1998, **158:** 1321-1324.

7. Miller E, Hare JW, Cloherty JP, Dunn PJ, Gleason RE, Soeldner JS *et al*.: **Elevated maternal hemoglobin A1c in early pregnancy and major congenital anomalies in infants of diabetic mothers.** *N Engl J Med* 1981, **304:** 1331-1334.

8. Hod M, van Dijk DJ, Karp M, Weintraub N, Rabinerson D, Bar J *et al*.: **Diabetic nephropathy and pregnancy: the effect of ACE inhibitors prior to pregnancy on fetomaternal outcome.** *Nephrol Dial Transplant* 1995, **10:** 2328-2333.

9. Jouatte F, Aitken B, Dufour P, Valat AS, Vamberghe A, Cappoen JP *et al*.: **Diabetes prior to pregnancy: 143 cases.** *Contraception Fertilite Sexualite* 1999, **27:** 845-852.

10. Rowe BR, Barnett AH: **Pre-conception counselling in Asian women with non insulin dependent diabetes and impaired glucose tolerance.** *Diabetes Res* 1988, **8:** 35-38.

11. McElvy SS, Miodovnik M, Rosenn B, Khoury JC, Siddiqi T, Dignan PS *et al*.: **A focused preconceptional and early pregnancy program in women with type 1 diabetes reduces perinatal mortality and malformation rates to general population levels.** *J Matern Fetal Med* 2000, **9:** 14-20.

12. Pearson DWM, Kernaghan D, Lee R, Penney GC: **The relationship between pre-pregnancy care and early pregnancy loss, major congenital anomaly or perinatal death in type I diabetes mellitus.** *BJOG: An International Journal of Obstetrics and Gynaecology* 2007, **114:** 104-107.

13. Dicker D, Feldberg D, Karp M: **Preconceptional diabetes control in insulin-dependent diabetes mellitus patients with continuous subcutaneous insulin infusion therapy.** *Journal of Perinatal Medicine* 1987, **15:** 161-167.

14. Kinsley B: **Achieving better outcomes in pregnancies complicated by type 1 and type 2 diabetes mellitus.** *Clin Ther* 2007, **29 Suppl D:** S153-S160.

15. Rodgers BD, Rodgers DE: **Efficacy of preconception care of diabetic women in a community setting.** *J Reprod Med* 1996, **41:** 422-426.

16. Unger J: **Preconception Management of Women with Type 1 Diabetes.** *The Female Patient* 2001, **26:** 40-46.

17. Garcia A, Corcoy R, Albareda M, Caballero A, Adelantado J, Altirriba O *et al*.: **Diabetic pregnancy: Prepregnancy care and pregnancy outcome.** *Diabetologia* 1997, **40:** 879.

18. Forde R, Connolly C, Murray S, Byrne MM, Firth RG, Kinsley BT: **Benefits of attending a dedicted pre-pregnancy service for women with diabetes mellitus-preliminary findings.** *Irish Journal of Medical Science* 2006, **175:** 21.

19. Kitzmiller JL, Buchanan TA, Kjos S, Combs CA, Ratner RE: **Pre-conception care of diabetes, congenital malformations, and spontaneous abortions.** *Diabetes Care* 1996, **19:** 514-541.

20. Mathiesen ER, Kinsley B, Amiel SA, Heller S, McCance D, Duran S *et al*.: **Maternal glycemic control and hypoglycemia in type 1 diabetic pregnancy: a randomized trial of insulin aspart versus human insulin in 322 pregnant women.** *Diabetes Care* 2007, **30:** 771-776.

**Appendix 3**:

**Maternal and fetal outcomes not improved by preconception care**

**Macrosomia**

**Cesarean section**

**Pre-eclampsia**

**Neonatal Hypoglycemia**

**Spontaneous Abortion**

**Respiratory Distress Syndrome (RDS)**

**Small for Gestation Age (SGA)**
